# Ovarian hyperstimulation syndrome- an optimal solution for an unresolved enigma

**DOI:** 10.1186/1757-2215-6-77

**Published:** 2013-11-05

**Authors:** Raoul Orvieto

**Affiliations:** 1 Infertility and IVF Unit, Department of Obstetrics and Gynecology, Sheba Medical Center, Ramat Gan, Israel; 2 Faculty of Health Sciences, Ben Gurion University of the Negev, Beer Sheva, Israel

**Keywords:** Ovarian hyperstimulation syndrome, Ovarian stimulation, GnRH agonist trigger, Prediction, Prevention

## Abstract

Ovarian hyperstimulation syndrome (OHSS) is a serious complication of controlled ovarian hyperstimulation (COH). The syndrome almost always presents either after hCG administration in susceptible patients or during early pregnancy. Despite many years of clinical experience, there are no precise methods to completely prevent severe OHSS, except by withholding the ovulation-inducing trigger of hCG. Recently, COH which combining GnRH antagonist co-treatment and GnRH agonist trigger has become a common tool aiming to *eliminate* severe early OHSS. However, the observed decrease in implantation and pregnancy rates following this approach has encouraged different modifications of luteal support aiming to improve outcome. One of the suggest approach is the 1500 IU hCG luteal rescue, which appears to be a promising protocol, aiming to reduce (*rather than eliminating*) severe early OHSS, without compromising outcome. In the present paper we discuss the different suggested strategies and offer a strict triage, aimed at eliminating the occurrence of severe OHSS based on several clinical observations, including the role of GnRH-antagonist in COH protocols, the use of different luteal rescue protocols and the ability to transfer embryos in the blastocyst stage.

## Introduction

Ovarian hyperstimulation syndrome (OHSS) is a serious complication of ovulation induction, almost always presents either after hCG administration in susceptible patients or during early pregnancy. Its cardinal features are marked ovarian enlargement and an increase in capillary permeability, with the consequent acute third-space fluid sequestration and its related morbidity [1]. Despite many years of clinical experience, the pathophysiology of OHSS is poorly understood, there is no reliable test to predict patients who will subsequently develop severe OHSS [2] and there are no precise methods to completely prevent severe OHSS.

Ideally, assisted reproduction technology (ART) practitioners seek a balance between optimum ovarian stimulation and successful treatment outcome with minimal rate of severe OHSS or multiple pregnancies. Individualization of treatment according to the specific risk factor and the specific response in the current cycle with the option of freezing of all embryos, or replacement of only a single embryo, has the potential of reducing the risk and the severity of the syndrome in susceptible cases. Moreover, while withholding the ovulation-inducing trigger of hCG, or replacing hCG with GnRH agonist (GnRHa) to trigger ovulation [3], may *eliminate* severe early OHSS, these methods are associated with decrease reproductive outcome.

### GnRHa trigger

Controlled ovarian hyperstimulation (COH) which combines GnRH antagonist co-treatment and GnRHa trigger has recently become a common tool aiming to *eliminate* severe early OHSS and to support the concept of an OHSS-free clinic [4,5]. However, due to the reported significantly reduced clinical pregnancy and increased first trimester pregnancy loss [6,7], efforts have been made to improve reproductive outcome, by manipulating the luteal phase. While discussing the recent developments in GnRHa trigger, Kol and Humaidan [8] presented 3 optional strategies aiming to improve outcome: freeze-all policy; fresh transfer and intensive luteal support; and fresh transfer and low-dose HCG supplementation.

*Freeze-all policy* is offered in extreme cases [5] in an attempt to ensure OHSS risk-free and maintain a reasonable cumulative pregnancy rate [9]. However, despite the recent improvement in live birth rates after replacement of frozen-thawed vitrified oocytes/embryos, it should be emphasized, that in most centers, there is still a gap in live birth rates between fresh and frozen/thawed cycles (in favour of fresh cycle).

*Intensive luteal support of estradiol (E2) and progesterone*, as described by Engmann et al. [10]. The data regarding the efficacy of luteal phase rescue after GnRHa trigger followed by intensive luteal phase support are intriguingly conflicting. We compared our experience with GnRHa trigger before [7] and after modifying our luteal-phase support to the intensive support with E2 and progesterone similar to the one reported by Engmann et al. [10]. We could not demonstrated any differences in peak E2 levels, fertilization rate, number of embryos transferred or implantation and pregnancy rates, between the two luteal support regimens [11]. Of notice, that in both groups of luteal support following GnRH-a trigger, implantation and pregnancy rates were lower compared to HCG trigger [7].

*One bolus of 1500 IU hCG* 35 h after the triggering bolus of GnRHa, i.e. one hour after oocyte retrieval [10], was demonstrated to rescue the luteal phase, resulting in a reproductive outcome comparable with that of HCG triggering, and with no increased risk of OHSS [12,13]. Moreover, in their recent review on GnRHa triggering, Kol and Humaidan [8] have suggested, that once a decision to use GnRHa trigger has been made, the options of fresh transfer and low-dose HCG supplementation, should not be implemented to patients “at high risk to develop OHSS”, such as those with >25 follicles. In a recently published retrospective study, Seyhan et al [14] have challenged the safety of the GnRHa trigger with HCG rescue. They examined whether GnRHa trigger and 1500 IU hCG luteal rescue protocol, completely prevented severe OHSS. Of the 23 patients evaluated, 6 (26%) developed severe OHSS, 5 of whom had severe early OHSS requiring ascites drainage and hospitalization. Moreover, a closer look at the cycle characteristics of those who developed severe early OHSS, 4 out of 5 had ≤25 follicles- a *safe threshold* according to Kol and Humaidan [8]. Moreover, their peak E2 levels and the number of oocytes retrieved ranged between 2563 to 8364 pg/mL, and 23 to 65, respectively.

The crucial issue, to our opinion, is how to recognize patients at risk for OHSS, and whether >25 follicles is an accurate and reliable predictive measure to identify patients at risk for OHSS? The ideal strategy that will eliminate OHSS without compromising ART outcome is still under search.

### Prediction of OHSS

Navot et al. [15] have reviewed the epidemiological, hormonal, and ultrasonographic characteristics of patients prone to develop OHSS. These included, among others, patients with an excessively high E2 response (>4000-6000 pg/ml, in IVF cycles) on the day of hCG administration and with multiple (more than 35) small and intermediate follicles that will yield more than 30 oocytes on retrieval. These characteristics were further supported by Asch et al. [16] who demonstrated that the combination of E2 levels >6000 pg/ml on the day of hCG administration and more than 30 retrieved oocytes is associated with an 80% chance of developing severe OHSS, while an almost negligible risk of OHSS was observed in IVF cycles with serum E2 <3500 pg/ml and/or less than 20 oocytes obtained at follicular aspiration. More than a decade later, Papanikolaou et al. [17], while assessing predictive values for identifying patients at risk for OHSS, revealed that an optimal threshold of ≥13 follicles with a diameter of >11 mm (85.5% sensitivity; 69% specificity) was statistically significantly superior than E2 concentrations of 2,560 ng/L (53% sensitivity, 77% specificity), on day of HCG administration. Moreover, the combination of a threshold of ≥18 follicles and/or E2 of ≥5,000 ng/L yields a 83% sensitivity rate with a specificity as high as 84% for the severe OHSS cases [17].

Of emphasize, that while investigating randomized controlled trials that aimed to prevent of severe OHSS, we found that different studies defined high-risk patients by different E2 threshold levels, ranging from 1906 to 6000 pg/ml, most used a level of ~3000 pg/ml [18]. Moreover, when all the predictive variables for severe OHSS were combined, the prevalence of severe OHSS in the ostensibly high risk patients was only about *20%*[18]- an extremely low value for a good predictive measure (Detection rate with 80% false positive). This figure is comparable to the 26% prevalence of severe OHSS, observed in Seyhan study [14].

### Prevention of OHSS

Secondary prevention requires not only knowledge of the pathophysiological mechanisms of the disease and means of intervention to correct the pathophysiological changes, but also the availability of early detection methods [19]. Since studies of the prevention of severe OHSS have been limited by the low sensitivity and predictive values of the factors currently used to define high risk [20], secondary prevention is limited as well.

The key to preventing OHSS is experience with ovulation stimulation therapy and recognition of “risk factors” for OHSS. For high-responder patients undergoing their first IVF attempt, it would be prudent to perform ovarian stimulation with a GnRH antagonist which provides the option of substituting HCG with GnRH agonist to trigger ovulation, and thereby eliminating severe OHSS [4,7].

It is noteworthy, that by a strict adherence to our previously published triage [4,21], we have already been practicing an OHSS- Free clinic, for the last 8 years. However, for ensuring patient safety, we are clearly paying by decrease ART outcome [7]. The recent advents in the aforementioned modified methods of the luteal support, have prompted us to improve our triage, into a more strict, comprehensive and individually-tailored approach to patients triggered with GnRHa. An approach that will eliminate severe early OHSS, in one hand, and will provide a reasonable pregnancy rate, in the other.

### Elimination of OHSS

Since neither excessive E2 levels, nor the number of developing follicles are reliable predictors for the development of severe OHSS [22], a different prudent strategy should be implemented.

**Figure 1:**
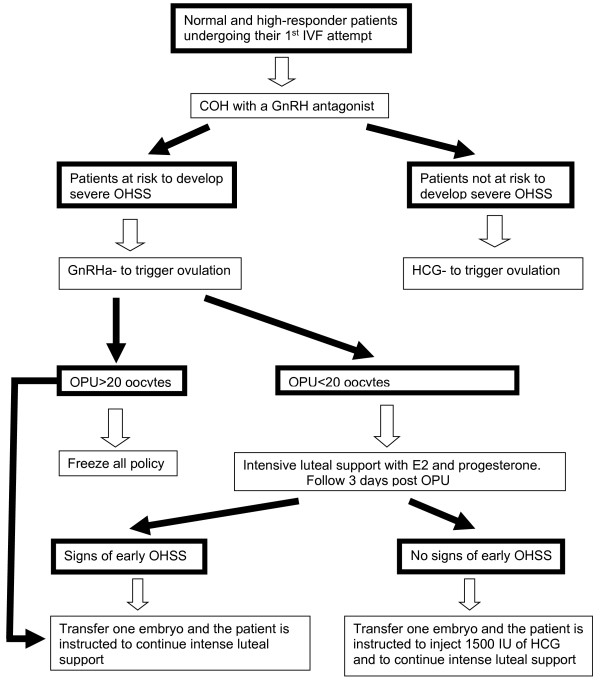
IVF treatment in normal and high-responder patients undergoing their first attempt.

In our practice, normal and high-responder patients undergoing their first IVF attempt are offered the COH with a GnRH antagonist (Figure 1). With this strategy we are able to substitute hCG with the GnRHa to trigger ovulation. GnRHa trigger if offered to patients at risk to develop severe OHSS. Those with rapidly rising serum E2 levels; Peak E2 level in excess of 2,500 pg/mL; and/or the emergence of a large number of intermediate sized follicles [23].

Following the aforementioned observation regarding GnRHa trigger with HCG rescue, we recently modified our approach to those triggered with GnRHa instead of HCG (Figure 1) [4]. In those achieving ≥20 oocytes, in accordance with the center’s freezing experience, the freeze all policy with the subsequent frozen-thawed embryo transfers (ET), or the transfer of one embryo with intense luteal support, are recommended. With the later, a lower pregnancy rate is expected with no case of severe OHSS.

If less than 20 oocytes are retrieved, patients are instructed to start an intensive luteal support with estradiol and progesterone, the day following OPU, and are re-evaluated 3 days after oocyte retrieval (day of ET) for signs of *early* moderate OHSS ((ultrasonographic signs of ascites as reflected by the appearance of fluid surrounding the uterus/ovaries, and/or Hct levels >40% for the degree of haemoconcentration). If signs of early moderate OHSS appear, one embryo is transferred, and the patient is instructed to continue with the intense luteal support. Of notice, lower pregnancy rates with no case of severe OHSS are expected [7,11].

However, if no early signs of OHSS developed, one embryo is transferred, and the patient is instructed to inject 1500 IU of HCG. By deferring the hCG bolus by 3 days (5 days following GnRHa trigger), we are still able to rescue the corpus luteum, with an observed extremely high midluteal progesterone levels (Orvieto, unpublished data). Moreover, we are actually offering the hCG to 80% of the *a priori* at risk patients (the aforementioned- detection rate c’ 80% false positive), who are not supposed to develop severe early OHSS, while avoiding hCG administration to the “real” 20-26% [14,18] patient at risk to develop severe early-OHSS. Despite a rather limited experience, no case of OHSS has been encountered.

**Figure 2:**
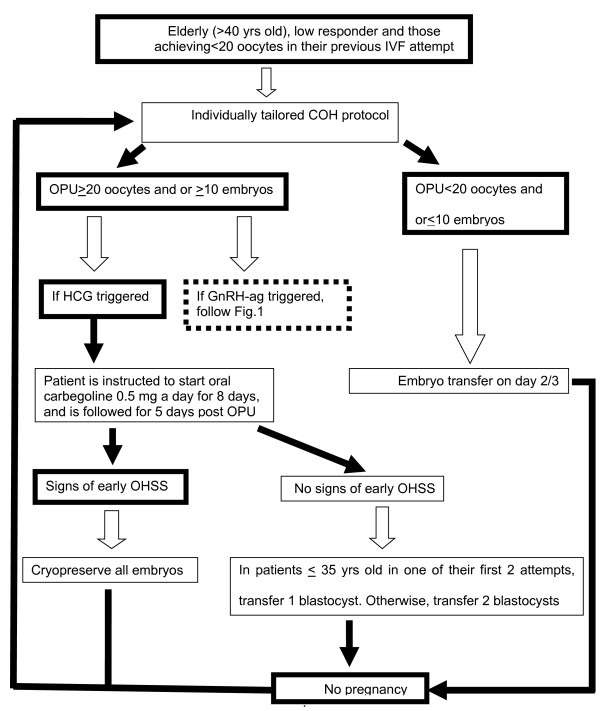
IVF treatment in elderly (>40 yrs), poor responders and patients in whom <20 oocytes were retrieved in the first IVF cycle attempt.

In patients in whom <20 oocytes were retrieved in the first IVF cycle attempt, and in low responders or patients >40 years old, the COH protocol is individually tailored (Figure 2). If the tailored COH protocols (with HCG trigger) yield ≥20 oocytes, or ≥10 embryos develop, the patient is instructed to start oral cabergoline 0.5 mg a day for 8 days [24], and is followed for 5 days after oocyte retrieval for signs of early OHSS (see above). If early signs develop, embryo transfer is withheld and all resulting embryos cryopreserved. This approach limits early OHSS, if it appears, to a milder and shorter form. If it does not appear, we transfer one blastocyst, with the consequent decrease in the risk of multiple pregnancy to almost zero, thereby eliminating the risk of late OHSS.

## Conclusion

COH using GnRH antagonist co-treatment and GnRHa trigger, combined with 1500 IU hCG luteal rescue, is a promising protocol, aiming to reduce (*rather than eliminating*) severe early OHSS, in on hand, and to improve reproductive outcome, in the other. However, in the meantime and until more studies elucidating the appropriate dose and timing of HCG administration will appear, strict adherence to the aforementioned strategy (Figures 1 and 2) is suggested.

## Abbreviations

ART: Assisted reproduction technology; COH: Controlled ovarian hyperstimulation; E2: Estradiol; GnRHa: GnRH agonist; OHSS: Ovarian hyperstimulation syndrome.

## Competing interests

The author declares that he has no competing interests.

## References

[B1] Navot D Bergh PA Laufer N Ovarian hyperstimulation syndrome in novel reproductive technologies: prevention and treatment Fertil Steril 1992 58 249 261 1633889 10.1016/s0015-0282(16)55188-7

[B2] Orvieto R Ben-Rafael Z Ovarian hyperstimulation syndrome: a new insight into an old enigma J Soc Gynecol Invest 1998 5 110 113 10.1016/S1071-5576(97)00113-5 9614638

[B3] Kol S Luteolysis induced by a gonadotropin-releasing hormone agonist is the key to prevention of ovarian hyperstimulation syndrome Fertil Steril 2004 81 1 5 14711532 10.1016/j.fertnstert.2003.05.032

[B4] Orvieto R Can we eliminate severe ovarian hyperstimulation syndrome? Hum Reprod 2005 20 320 322 15567876 10.1093/humrep/deh613

[B5] Devroey P Polyzos NP Blockeel C An OHSS-Free Clinic by segmentation of IVF treatment Hum Reprod 2011 6 2593 2597 21828116 10.1093/humrep/der251

[B6] Griesinger G Diedrich K Devroey P Kolibianakis EM GnRH agonist for triggering final oocyte maturation in the GnRH antagonist ovarian hyperstimulation protocol: a systematic review and meta-analysis Hum Reprod Update 2006 12 159 168 16254001 10.1093/humupd/dmi045

[B7] Orvieto R Rabinson J Meltzer S Zohav E Anteby E Homburg R Substituting HCG with GnRH agonist to trigger final follicular maturation–a retrospective comparison of three different ovarian stimulation protocols Reprod Biomed Online 2006 13 198 201 10.1016/S1472-6483(10)60615-3 16895632

[B8] Kol S Humaidan P GnRH agonist triggering: recent developments Reprod Biomed Online 2013 26 226 230 10.1016/j.rbmo.2012.11.002 23337420

[B9] Griesinger G Schultz L Bauer T Broessner A Frambach T Kissler S Ovarian hyperstimulation syndrome prevention by gonadotropin-releasing hormone agonist triggering of final oocyte maturation in a gonadotropin-releasing hormone antagonist protocol in combination with a 'freeze-all’ strategy: a prospective multicentric study Fertil Steril 2011 95 2029 2033 10.1016/j.fertnstert.2011.01.163 21371705

[B10] Engmann L DiLuigi A Schmidt D Nulsen J Maier D Benadiva C The use of gonadotropin-releasing hormone (GnRH) agonist to induce oocyte maturation after cotreatment with GnRH antagonist in high-risk patients undergoing in vitro fertilization prevents the risk of ovarian hyperstimulation syndrome: a prospective randomised controlled study Fertil Steril 2008 89 84 91 10.1016/j.fertnstert.2007.02.002 17462639

[B11] Orvieto R Intensive luteal-phase support with oestradiol and progesterone after GnRH-agonist triggering: does it help? Reprod Biomed Online 2012 24 680 681 10.1016/j.rbmo.2012.03.005 22503268

[B12] Humaidan P Bredkjaer HE Westergaard LG Andersen CY 1,500 IU human chorionic gonadotropin administered at oocyte retrieval rescues the luteal phase when gonadotropin- releasing hormone agonist is used for ovulation induction: a prospective, randomized, controlled study Fertil Steril 2010 93 847 854 10.1016/j.fertnstert.2008.12.042 19200959

[B13] Humaidan P Papanikolaou EG Kyrou D Alsbjerg B Polyzos NP Devroey P Fatemi HM The luteal phase after GnRH-agonist triggering of ovulation: present and future perspectives Reprod Biomed Online 2012 24 134 141 10.1016/j.rbmo.2011.11.001 22197130

[B14] Seyhan A Ata B Polat M Son WY Yarali H Dahan MH Severe early ovarian hyperstimulation syndrome following GnRH agonist trigger with the addition of 1500 IU hCG Hum Reprod 2013 28 9 2522 2528 10.1093/humrep/det124 23633553

[B15] Navot D Bergh PA Laufer N Adashi E, Rock JA, Rosenwaks Z The ovarian hyperstimulation syndrome Reproductive Endocrinology, Surgery, and Technology 1996 Philadelphia: Lippincott–Raven Publishers 2215 2232

[B16] Asch RH Lo HP Balmaceda JP Weckstein LN Stone SC Severe ovarian hyperstimulation syndrome in assisted reproductive technology: defnition of high risk groups Hum Reprod 1991 6 1395 1399 1770133 10.1093/oxfordjournals.humrep.a137276

[B17] Papanikolaou EG Pozzobon C Kolibianakis EM Camus M Tournaye H Fatemi HM Van Steirteghem A Devroey P Incidence and prediction of ovarian hyperstimulation syndrome in women undergoing gonadotropin-releasing hormone antagonist in vitro fertilization cycles Fertil Steril 2006 85 112 120 10.1016/j.fertnstert.2005.07.1292 16412740

[B18] Orvieto R Ben-Rafael Z Role of intravenous albumin in the prevention of severe ovarian hyperstimulation syndrome Hum Reprod 1998 13 3306 3309 10.1093/humrep/13.12.3306 9886504

[B19] Orvieto R Achiron A Ben-Rafael Z Hagay Z Achiron R The possible role of intravenous immunoglobulin in preventing preeclampsia Med Hypoth 1993 41 160 164 10.1016/0306-9877(93)90063-V 8231996

[B20] Levy T Orvieto R Homburg R Dekel A Peleg D Ben-Rafael Z Severe ovarian hyperstimulation syndrome despite low plasma estrogen levels in a hypogonadotropic hypogonadal patient Hum Reprod 1996 11 1177 1179 10.1093/oxfordjournals.humrep.a019350 8671418

[B21] Orvieto R Elimination of ovarian hyperstimulatin syndrome Fertil Steril 2012 97 e29 10.1016/j.fertnstert.2012.03.019 22503420

[B22] Orvieto R Prediction of OHSS—challenging the estradiol mitos Hum Reprod 2003 18 665 667 10.1093/humrep/deg166 12660254

[B23] The Practice Committee of the American Society for Reproductive Medicine (ASRM) Ovarian hyperstimulation syndrome Fertil Steril 2008 90 S188 S193 19007627 10.1016/j.fertnstert.2008.08.034

[B24] Soares SR Etiology of OHSS and use of dopamine agonists Fertil Steril 2012 97 517 522 10.1016/j.fertnstert.2011.12.046 22265002

